# Expectant treatment for angular pregnancy after assisted reproduction technology: a safe and patient-friendly treatment strategy

**DOI:** 10.3389/fmed.2023.1234425

**Published:** 2023-08-22

**Authors:** Peiwen Yang, Lin Shen, Jihui Ai, Yiqing Zhao

**Affiliations:** Reproductive Medicine Center, Department of Gynecology and Obstetrics, Tongji Hospital, Tongji Medical College, Huazhong University of Science and Technology, Wuhan, China

**Keywords:** angular pregnancy, expectant treatment, live birth, term birth, assisted reproductive technology

## Abstract

**Introduction:**

Currently, the treatment strategies for angular pregnancy in the first trimester after assisted reproduction technology (ART) are unclear. Improper treatment will cause unnecessary losses to patients, especially infertile patients, after ART. The purpose of this study was to clarify the pregnancy outcomes of expectant treatment for angular pregnancy post-ART and to provide a basis for the formulation of clinical treatment strategies.

**Method:**

This retrospective case series study was performed at the Reproductive Medicine Center of a university hospital. Maternal data and pregnancy outcomes were collected and analyzed for all patients diagnosed with angular pregnancies after ART between January 2016 and August 2021. The outcomes included live birth, term birth, premature birth, early pregnancy loss, fetal death, placental abruption, uterine rupture, maternal death, and hysterectomy.

**Results:**

A total of 78 patients were analyzed in this study, of whom 54 (69.2%) had live births, 44 (56.4%) had term births, 21 (26.9%) had an early pregnancy loss, 1 (1.3%) had mid-trimester missed abortion, 1 (1.3%) underwent mid-trimester labor induction due to fetal malformation, and 1 (1.3%) underwent uterine rupture. There were no cases of maternal death, placental abruption, or hysterectomies.

**Discussion:**

Angular pregnancy after ART is not as dangerous as that described in previous studies; most cases could be treated expectantly under close-interval follow-up and obtain live birth.

## 1. Introduction

After receiving assisted reproductive technology (ART), some patients are diagnosed with an angular pregnancy during regular ultrasound examinations. Angular pregnancy was originally defined by Dr. Howard Kelly in 1898 as the implantation of the embryo just medial to the uterotubal junction at the lateral angle of the uterine cavity (1). Although the term angular pregnancy has existed for more than a century, the diagnosis and treatment of angular pregnancy are still challenging due to a lack of clinical experience in this subject as well as a paucity of published literature addressing the diagnosis and management of angular pregnancy (2).

Given the limited literature on this rare condition, the management of angular pregnancy remains controversial. Several studies have reported severe outcomes in angular pregnancy (3, 4). Based on this evidence, angular pregnancies are considered high-risk pregnancies in relation to spontaneous abortion, uterine rupture, and maternal death. And as a result, the management of angular pregnancies overwhelmingly involved pregnancy termination and laparoscopy surgery (3, 4).

Recently, with the use of ultrasonography in the evaluation of early pregnancy, asymptomatic angular pregnancies have been detected early in the first trimester. Research published by Kassie J. Bolling’s developed five criteria for diagnosing angular pregnancies (5). It showed that the majority of patients with angular pregnancy had a live birth, indicating angular pregnancy as a non-eccentric pregnancy rather than an ectopic pregnancy (5). However, uterine rupture, which can lead to severe intra-abdominal hemorrhage and consequent maternal death, has occasionally been reported (6, 7).

Considering the risk of spontaneous abortion, uterine rupture, and maternal death, so much so that some clinicians will recommend termination of pregnancy at an early stage (8–12). Patients with infertility spend considerable time and money on ART. Therefore, termination of pregnancy due to angular pregnancy may have caused great loss in this patient. Treatment of these patients remains a dilemma for clinicians. To solve this problem, this study describes the pregnancy outcomes of angular pregnancy cases after receiving ART that was managed expectantly after early diagnosis in the first trimester. We hope that our study will help clinicians develop appropriate treatment strategies.

## 2. Materials and methods

### 2.1. Study design

**Figure 1:**
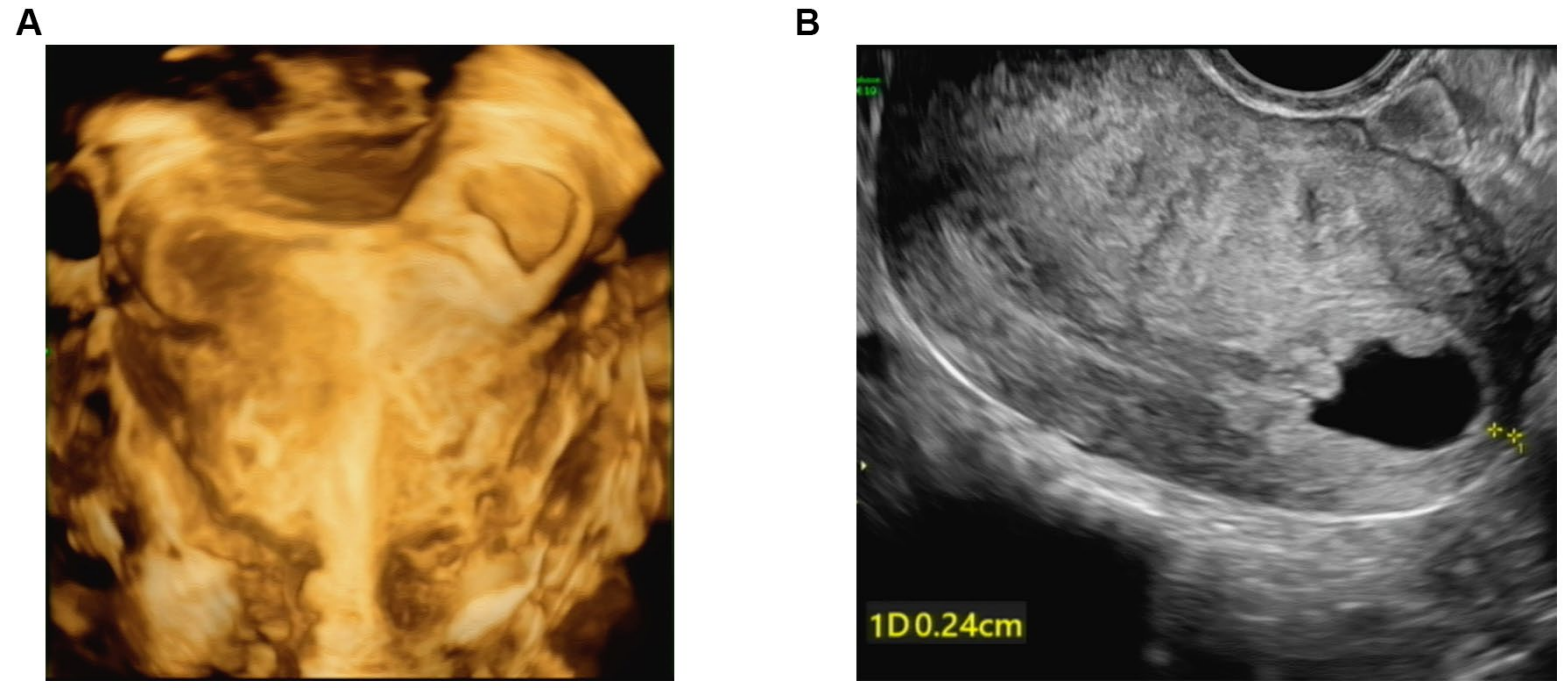
Ultrasonography images of angular pregnancy. **(A)** Three-dimensional ultrasonography image of an angular pregnancy with implantation medial to the uterotubal junction. **(B)** Two-dimensional ultrasonography image of an angular pregnancy with a myometrial thickness of 0.24 cm.

This retrospective case series study was approved by the Institutional Review Board of the Tongji Hospital (TJ-IRB20211007). Medical records and ultrasound reports were reviewed for all patients diagnosed with angular pregnancies who were treated expectantly after ART between January 2016 and 2021 at the Reproductive Medicine Center, Tongji Hospital, Tongji Medical College, Huazhong University of Science and Technology. All patients were offered transvaginal ultrasound scans during the first trimester of pregnancy. The diagnostic criteria are as follows:(1) Implantation of the embryo in the lateral angle of the uterine cavity, just medial to the uterotubal junction (5); (2) No more than 0.5 cm of myometrial thickness from the gestational sac to the outer border of the uterus (Figure 1); (3) Presence of completely circumferential endometrium surrounding the gestational sac and, therefore, diagnostic of intrauterine gestation (5); (4) Lack of an ‘interstitial line sign.’ (5) Patients with an anomalous uterus, such as a unicornuate, bicornuate, or septate, were excluded (5). Furthermore, cases in which angular pregnancies were artificially terminated immediately after diagnosis in the absence of any symptoms or indications were excluded.

The collected maternal data included age (years), gravidity, parity, height, weight, body mass index (BMI, calculated as weight in kilograms divided by height in meters squared), number of gestational sacs, maternal medical conditions (including systemic diseases/factors, gynecological diseases/factors, and obstetric diseases), symptoms (such as abdominal pain, vaginal bleeding, and hemometra), myometrial thickness, and gestational age at initial diagnosis, and the thinnest myometrial thickness and its gestational age.

The final pregnancy outcomes included live birth, term birth, premature birth, early pregnancy loss, fetal death, placental abruption, uterine rupture, maternal death, and hysterectomy.

### 2.2. Statistical analysis

Means and standard deviations were used to describe continuous variables and frequencies and percentages were used to describe categorical variables. Student’s *t*-tests were used to compare continuous variables. For proportional differences and categorical outcomes, χ2 - tests were applied for trends statistic, and if the difference was significant, the odds ratio (OR) and 95% confidence interval (CI) compared with the lowest group were calculated. Measures concerning scale variables were compared using the one-way analysis of variance (ANOVA) method. Finally, factors related to clinical pregnancy rate were assessed using multivariate logistic regression analysis. Statistical analyses were performed using SPSS 23.0 statistical analysis software for Windows (IBM). All tests were two-sided and were considered significant at *p* < 0.05.

## 3. Results

**Figure 2:**
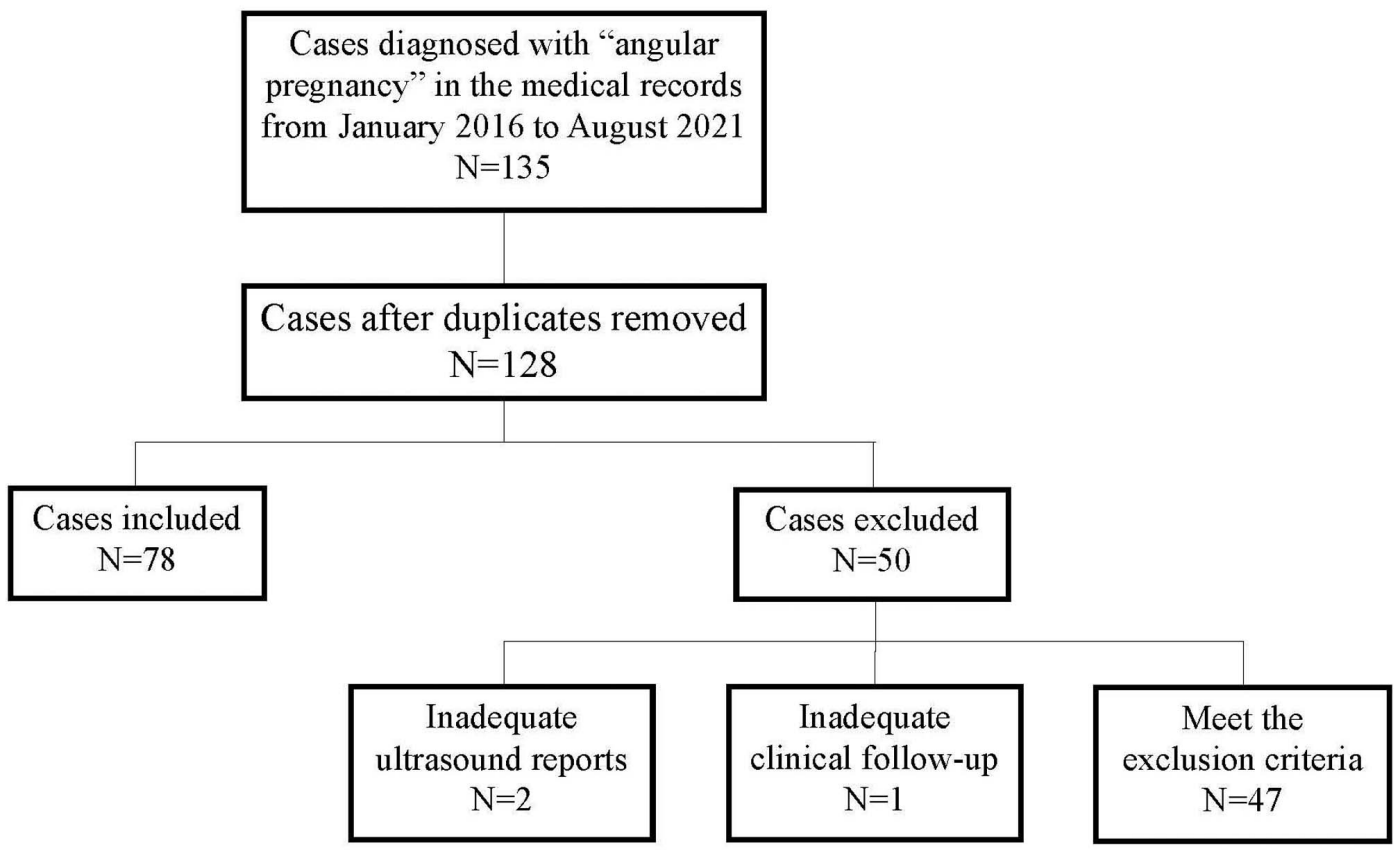
Inclusion and exclusion of angular pregnancy cases.

As shown in Figure 2, 135 patients were diagnosed with angular pregnancies based on their medical records. Seven duplicate cases were excluded; three cases were excluded due to inadequate ultrasound reports (*N* = 2) or clinical follow-up (*N* = 1) and 47 cases were excluded according to the exclusion criteria. Finally, 78 cases were included in the study.

Baseline maternal characteristics are shown in Table 1. The maternal conditions are presented in Table 2. Of all the 78 patients, 16 (20.5%) had systemic diseases/factors, including advanced maternal age (age ≥ 35 years), polycystic ovary syndrome, hyperthyroidism/hypothyroidism, insulin resistance, and type 2 diabetes mellitus. The gynecologic and obstetric diseases/factors affecting pregnancy are summarized in Table 2. Fifty-seven of the 78 patients (73.1%) had a history of pelvic surgery and/or uterine surgery. Twenty (25.6%) patients had gynecologic diseases, including intrauterine adhesions, uterine myomas, adenomyosis, endometrial tuberculosis, and cervical cancer. Obstetric diseases were observed in 9 (11.5%) patients during their pregnancies, including gestational diabetes mellitus, gestational hypertension, and placenta previa.

**Table 1:** Baseline maternal characteristics.

Characteristics	Patients (*N* = 78)
Age, y	30.4 ± 3.7 (29.6–31.3)
BMI, kg/m^2^	22.1 ± 3.4 (21.4–22.9)
Gravidity	2 (1–5)
Parity	0 (0–1)
Gestational sacs, no.	1.3 ± 0.5 (1.8–2.3)

BMI, body mass index.

Data are presented as the mean ± standard dviation (95% confidence interval), or median (range).

**Table 2:** Maternal medical conditions.

Maternal medical conditions	*n* (% of all cases) [95% CI]
Systemic diseases/factor	
Advanced maternal age	11 (14.1) [8.1–23.5]
Polycystic ovary syndromes	2 (2.6) [0.7–8.9]
Hyperthyroidism/Hypothyroidism	3 (3.8) [1.3–10.7]
Insulin resistance	2 (2.6) [0.7–8.9]
Type 2 diabetes mellitus	1 (1.3) [0.2–6.9]
Gynecologic diseases/factor	
Intrauterine adhesion	4 (5.1) [2.0–12.5]
Uterine myoma	13 (16.7) [10.0–26.5]
Adenomyosis	4 (5.1) [2.0–12.5]
Endometrial tuberculosis	2 (2.6) [0.7–8.9]
Cervical cancer	1 (1.3) [0.2–6.9]
Surgical history	
Prior pelvic surgery	33 (42.3) [32.0–53.4]
Prior uterine surgery	42 (53.8) [44.1–65.7]
Prior pelvic and uterine surgery	18 (23.1) [15.1–33.6]
Obstetric diseases	
Gestational diabetes mellitus	5 (6.4) [2.7–14.1]
Gestational hypertension	2 (2.6) [0.7–8.9]
Placenta previa	4 (5.1) [2.0–12.5]

CI, confidence interval.

The clinical characteristics of angular pregnancies are shown in Table 3. More than half of the patients (55.1%) had symptoms such as vaginal bleeding, hematometra, or hypogastria. When patients were initially diagnosed with angular pregnancy, the mean myometrial thickness was 0.30 ± 0.11 cm (95% CI 0.27–0.32), and the mean gestational age was 8.0 ± 1.2 weeks (95% CI 77.8–8.3). The mean thinnest myometrial thickness during pregnancy was 0.26 ± 0.10 cm (95% CI 0.24–0.28), and the mean gestational age was 8.6 ± 1.5 weeks (95% CI 8.2–8.9).

**Table 3:** Clinical characteristics of angular pregnancy.

Clinical characteristics	Patients (*N* = 78)
Symptomatic, *n* (%)	42 (55.1) [44.1–65.7]
Myometrial thickness(initial) (cm)	0.30 ± 0.11 [0.27–0.32]
Myometrial thickness(initial), *n* (%)	
<0.1 cm	3 (3.8) [1.3–10.7]
0.1–0.2 cm	6 (7.7) [3.6–15.8]
0.2–0.3 cm	28 (35.9) [26.2–47.0]
0.3–0.4 cm	20 (25.6) [17.3–36.3]
0.4–0.5 cm	21 (26.9) [18.3–37.7]
Gestational age (initial) (week)	8.0 ± 1.2 [7.8–8.3]
Myometrial thickness (thinnest) (cm)	0.26 ± 0.10 [0.24–0.28]
Myometrial thickness (thinnest), *n* (%)	
<0.1 cm	3 (3.8) [1.3–10.7]
0.1–0.2 cm	10 (12.8) [7.1–22.0]
0.2–0.3 cm	40 (51.3) [40.4–62.1]
0.3–0.4 cm	13 (16.7) [10.0–26.5]
0.4–0.5 cm	12 (15.4) [9.0–25.0]
Gestational age (thinnest) (week)	8.6 ± 1.5 [8.2–8.9]

Data are presented as the mean ± standard deviation [95% confidence interval], or *n* (%) [95% confidence interval].

The outcomes of angular pregnancies are summarized in Table 4. Fifty-four (69.2%) deliveries resulted in live births. Of these live births, 44 (56.4%) were full-term births and 10 (12.8%) were premature births. Notably, the thinnest myometrial thickness resulting in a term birth was 0.14 cm. The 24 cases that did not result in live births included 21 (26.9%) early pregnancy losses, 1 (1.3%) mid-trimester missed abortion, 1 (1.3%) mid-trimester labor induction because of fetal malformation, and 1 (1.3%) uterine rupture. There were no cases of maternal death, placental abruption, or hysterectomies.

**Table 4:** Overall outcomes of angular pregnancies (*n* = 78).

Pregnancy outcomes	*n* (% of all cases) [95% CI]
Live birth	54 (69.2) [58.3–78.4]
Singleton (*n* = 40)	
Twins (*n* = 14)	
Term birth	44 (56.4) [45.3–66.9]
Premature birth	10 (12.8) [7.1–22.0]
Early pregnancy loss	21 (26.9) [18.3–37.7]
Mid-trimester missed abortion	1 (1.3) [0.2–6.9]
Mid-trimester labor induction due to fetal malformation	1 (1.3) [0.2–6.9]
Uterine rupture	1 (1.3) [0.2–6.9]

Data are presented as n (%) [95% confidence interval].

Uterine rupture occurred in a 31 year-old woman (gravida 5, para 1) with a history of two laparoscopic surgeries (tubal pregnancy, right salpingectomy), one cesarean delivery and one artificial abortion. She was treated with *in vitro* fertilization and embryo transfer because of infertility and underwent third-day embryo transfer. Routine luteal support was provided on the day of embryo transfer. At 14 days after transfer, a blood test to evaluate human chorionic gonadotropin was performed and revealed a level of 550.7mIU/mL. The ultrasound examination performed at another clinic at 5 weeks of gestation revealed a gestational sac located at the left angle of the uterus with a myometrial thickness of 0.2 cm. The patient was asymptomatic, therefore, she continued her pregnancy with close monitoring. However, she developed lower abdominal pain at 6 weeks of gestation. When she arrived at the hospital, an emergency B-ultrasound examination was performed. It revealed a mixed echogenic mass at the left angle of the uterus and pelvic and abdominal fluid accumulation indicating hemoperitoneum. Emergency laparoscopic surgery was performed immediately, and rupture with protrusion of gestational tissue was found at the left uterine angle. We removed the gestational tissue and repaired the rupture. The postoperative course was uneventful.

Clinical features according to live births are summarized in Table 5. The thinnest myometrial thickness and myometrial thickness, when initially diagnosed in cases that achieved live births, were significantly greater in than those that did not. However, the gestational age (first and thinnest), advanced maternal age, prior pelvic surgery, prior uterine surgery, uterine disease, and symptoms were comparable between the two groups.

**Table 5:** Clinical features of live births.

	Live birth (*n* = 54)	No live birth (n = 24)	*p* value
Myometrial thickness (initial) (cm)	0.32 ± 0.09	0.25 ± 0.13	0.006*
Myometrial thickness (initial), *n* (%)			
<0.1 cm	0 (0%)	3 (12.5%)	
0.1–0.2 cm	4 (7.4%)	2 (8.3%)	
0.2–0.3 cm	16 (29.6%)	12 (50.0%)	
0.3–0.4 cm	17 (31.5%)	3 (12.5%)	
0.4–0.5 cm	17 (31.5%)	4 (16.7%)	
Gestational age (initial) (week)	8.0 ± 1.1	8.2 ± 1.4	0.475
Myometrial thickness (thinnest) (cm)	0.28 ± 0.08	0.22 ± 0.12	0.014*
Myometrial thickness (thinnest), *n* (%)			
<0.1 cm	0 (0%)	3 (12.5%)	
0.1–0.2 cm	6 (11.1%)	4 (16.7%)	
0.2–0.3 cm	28 (51.9%)	12 (50.0%)	
0.3–0.4 cm	11 (20.4%)	2 (8.3%)	
0.4–0.5 cm	9 (16.7%)	3 (12.5%)	
Gestational age (thinnest) (week)	8.6 ± 1.5	8.5 ± 1.7	0.505
Advanced maternal age (≥ 35 years), *n* (%)	6 (11.1%)	5 (20.8%)	0.255
Prior pelvic surgery, *n* (%)	22 (40.7%)	11 (45.8%)	0.674
Prior uterine surgery, *n* (%)	29 (53.7%)	13 (54.2%)	0.970
Uterine diseases, *n* (%)	12 (22.2%)	7 (29.2%)	0.510
Symptoms, *n* (%)	29 (53.7%)	14 (58.3%)	0.704

Uterine diseases included intrauterine adhesion, uterine myoma, adenomyosis, and endometrial tuberculosis.

* Statistically significant.

## 4. Discussion

Although angular pregnancy was defined and discussed in 1989 (1), its treatment remains controversial. Improper treatment will cause unnecessary losses to patients, especially infertile patients, after ART. Angular pregnancy involves an aberrant implantation site that leads to spontaneous abortion, uterine rupture, and maternal death (3, 4). However, as angular pregnancy occurs within the endometrial cavity, patients with angular pregnancy can achieve full-term delivery (13).

In 1981, Jason and Elliot used laparoscopy to define angular pregnancies and set specific criteria (4). In studies using these criteria, angular pregnancies were mainly diagnosed in patients with severe clinical symptoms and were detected largely in the later second and third trimesters. Since asymptomatic patients might be undiagnosed, outcomes might be biased toward a bad situation, and the proportion of normally progressed cases that resulted in live births could be underestimated (5). Moreover, the term angular pregnancy has been used interchangeably with interstitial pregnancy; the latter is considered non-viable, and the termination of pregnancy is proposed as a lifesaving treatment (14, 15). Therefore, angular pregnancies were regarded as high-risk pregnancies, and pregnancy termination and laparoscopic surgery were advised and performed in the majority of studies (16).

Pregnancy is difficult in patients with infertility. Some patients require ART assistance. To obtain a pregnancy, a lot of time and money are spent, and suffer a great deal of mental stress. According to most studies, women are advised to terminate their pregnancy when they are diagnosed with an angular pregnancy. It will be a great loss and blow to them, both mentally and financially. Furthermore, whether and when they can achieve their next pregnancy is uncertain. Therefore, it is very important to determine whether angular pregnancy is a high-risk pregnancy and whether it is necessary to terminate it. In recent years, with the development of ultrasonographic criteria for the diagnosis of angular pregnancy, more angular pregnancies, especially in asymptomatic patients, have been detected. Some patients diagnosed early were treated expectantly, resulting in live births (14, 17, 18).

In our study, all the patients with angular pregnancies underwent expectant management. Our data showed that the live birth rate was 69.2%, the early pregnancy loss rate was 29.2%, and the uterine rupture rate was 1.3% (only one patient), without maternal death, placental abruption, or hysterectomy. These results suggest that angular pregnancy is not as dangerous as previously described. Our results contradicted those of previous studies. Using transvaginal ultrasonography in the first trimester may have resulted in the inclusion of asymptomatic patients in our study. Furthermore, patients with anomalous uteri such as unicornuate, bicornuate, and septate uteri were excluded. This allowed us to investigate the natural history of angular pregnancies after ART. Therefore, in most cases, expectant treatment can be performed under close monitoring.

Recently, a prospective study included 42 angular pregnancy cases that were diagnosed in the first trimester using specific ultrasound criteria. Expectant management was performed, and the outcomes of these pregnancies were followed (5). It has been reported that 80% of these pregnancies result in live births and 20% result in early pregnancy loss. There were no cases of uterine rupture, maternal death, abnormal placentation, or hysterectomies. These results are consistent with those of this study. The ultrasonographic criteria used in our study were based on those proposed by Kassie et al. (5) in 2020. One of our criteria was that the myometrial thickness from the gestational sac to the outer border of the uterus ≤0.5 cm, not 1 cm in the criteria of Kassie et al. The reason why we chose myometrial thickness ≤ 0.5 cm as a standard was that we want to figure out the pregnancy outcome of angular pregnancy under this condition. Previous studies have attempted to elucidate the relationship between myometrial thickness and pregnancy outcomes in angular pregnancies. In 2011, a case report showed that thin myometrium (3 mm) could result in a live birth (17). Another case in 2018 reported a term delivery with a thin myometrial thickness of 2–5 mm (14). The case series of Kassie et al. in 2020 did not consider myometrial thickness as a distinguishing or prognostic factor because the case in their study with the thinnest initial myometrial thickness of 2.3 mm resulted in term delivery, but the case with an initial myometrial thickness of 9.8 mm had a thinner myometrium on continued follow-up and eventually resulted in preterm delivery (5). In our study, a myometrial thickness of 0.14 cm resulted in live births, and there was only one case of uterine rupture with a myometrial thickness of 0.2 cm. Even if the myometrium is very thin, angular pregnancies can still be treated expectantly with a low risk of uterine rupture. However, our study also found that both the initial and thinnest myometrial thicknesses of patients without live births were thinner than those with live births, which indicated that adverse outcomes were more likely to occur in patients with thinner myometrium; therefore, more frequent follow-up was needed for those patients.

In 2020, Kassie et al. reported nine symptomatic patients with angular pregnancy, six of whom had live births (5). Our study had similar results, with the vast majority of patients having live births, regardless of whether they had symptoms. Therefore, for angular patients with symptoms, we recommend continuing the pregnancy with active symptomatic treatment and close follow-up. Patients with infertility usually have other factors that adversely affect pregnancy outcomes, including maternal age, history of pelvic or uterine surgery, and uterine diseases. Therefore, we analyzed these adverse factors in the present study. Our results showed that there was no significant difference in these factors between angular patients who had live births and those who did not. Therefore, even if patients with angular pregnancy had these adverse factors, they can be treated with expectation under close monitoring.

In conclusion, pregnancy is difficult in patients with infertility. Since angular pregnancy is not as dangerous as suggested in previous literature, the immediate termination of pregnancy is not recommended when patients are diagnosed with angular pregnancy after ART in the first trimester. Thus, we can try our best to help such patients and avoid huge economic and spiritual losses. The vast majority of patients with angular pregnancy after ART can achieve live births during close-interval follow-up, even if there are adverse factors affecting pregnancy outcomes in these patients.

## Data availability statement

The original contributions presented in the study are included in the article/supplementary material, further inquiries can be directed to the corresponding authors.

## Ethics statement

The studies involving humans were approved by the Institutional Review Board of the Tongji Hospital. The studies were conducted in accordance with the local legislation and institutional requirements. Written informed consent for participation was not required from the participants or the participants’ legal guardians/next of kin in accordance with the national legislation and institutional requirements.

## Author contributions

YZ funded the study. YZ and PY designed the study and wrote the manuscript. PY and LS performed all the investigations under the guidance of JA and YZ. JA and YZ supervised the experiments and data analysis. All authors discussed the data and reviewed the manuscript.

## Funding

This work was supported by the Basic Research Project for Young and Middle-Aged Physicians of the Beijing Health Promotion Association (BJHPA-2022-SHZHYXZHQNYJ-JICH-002) and the Scientific Research Starting Foundation for Returned Overseas Scholars (2020HGRY004) of Tongji Hospital.

## Acknowledgments

We would like to thank Editage (www.editage.com) for English language editing.

## Conflict of interest

The authors declare that the research was conducted in the absence of any commercial or financial relationships that could be construed as a potential conflict of interest.

## Publisher’s note

All claims expressed in this article are solely those of the authors and do not necessarily represent those of their affiliated organizations, or those of the publisher, the editors and the reviewers. Any product that may be evaluated in this article, or claim that may be made by its manufacturer, is not guaranteed or endorsed by the publisher.
